# The effect of exercise on the adverse neonatal outcomes related to women with gestational diabetes mellitus: a systematic review and meta-analysis

**DOI:** 10.3389/fcdhc.2025.1566577

**Published:** 2025-04-01

**Authors:** Hangyu Cui, Hua Li, Jing Huang, Yi Wu, Yuan Wei, Mingzi Li

**Affiliations:** ^1^ School of Nursing, Peking University, Beijing, China; ^2^ Department of Obstetrics and Gynecology, Peking University Third Hospital, Beijing, China

**Keywords:** diabetes, gestational, physical activity, fetus, pregnancy outcome, meta-analysis

## Abstract

This meta-analysis aimed to evaluate the benefits of prenatal exercise on neonatal outcomes in women with gestational diabetes mellitus (GDM). Systematic searches were conducted in PubMed, EMBASE, Cochrane Library, Web of Science, and Scopus from their inception to September 9, 2023. ClinicalTrials.gov was also searched to ensure comprehensive coverage. We included studies that investigated the association between prenatal exercise and at least one adverse neonatal outcome of interest. A total of 4,268 publications were retrieved, and 3,060 records remained after removing duplicates. After screening abstracts, 107 studies were selected for full-text assessment, and ultimately, 17 articles (including 4 identified through manual searching) were included for data extraction. Extracted information included the first author, publication year, study design, geographical location, sample size, participants’ demographic characteristics, intervention characteristics, and relevant outcome variables.Pooled results from random-effects models showed that prenatal exercise significantly reduced the risk of adverse neonatal outcomes, including: Cesarean delivery (OR = 0.91, 95% CI: 0.88–0.94), Premature birth (OR = 0.49, 95% CI: 0.27–0.90), Macrosomia (OR = 0.58, 95% CI: 0.40–0.83), Fetal growth restriction (OR = 0.21, 95% CI: 0.08–0.52), and Birth trauma (OR = 0.26, 95% CI: 0.13–0.54). Subgroup analyses indicated that single-component exercise programs were more effective than multi-component programs in reducing the risk of macrosomia (P = 0.06). In conclusion, prenatal exercise substantially reduces the risk of multiple adverse neonatal outcomes in women with GDM, including macrosomia, preterm birth, cesarean delivery, fetal growth restriction, and birth trauma. These findings highlight the outstanding benefits of antenatal exercise for fetal health, supporting its inclusion as a key component of prenatal care for women with GDM. This meta-analysis is registered with PROSPERO (Registration Number: CRD42023485375).

## Introduction

Gestational diabetes mellitus (GDM), defined as glucose intolerance with onset or first recognition during pregnancy, is one of the most common complications among pregnant women. Depending on the population and diagnostic criteria used, the prevalence of GDM ranges from 1% to over 30%, posing a significant global public health challenge (1–3). Women with GDM are at an elevated risk of experiencing other pregnancy complications, as well as developing type 2 diabetes, cardiovascular and cerebrovascular diseases, and metabolic syndrome later in life (4–6). Maternal hyperglycemia leads to fetal hyperglycemia through facilitated glucose transport mediated by glucose transporter 1 (GLUT1) (7). This fetal hyperglycemia triggers hyperinsulinemia, which promotes excessive fetal adiposity and accelerated growth, resulting in macrosomia and a range of associated complications, including preterm birth, cesarean delivery, and birth trauma (8–10). Poor maternal glucose control further exacerbates the risk of adverse pregnancy outcomes (10). Additionally, offspring of mothers with GDM are more likely to develop long-term metabolic and cardiovascular disorders (11). Given these risks, timely and effective interventions to mitigate adverse pregnancy and neonatal outcomes associated with GDM, along with continuous monitoring of maternal and child health postpartum, are of critical clinical importance (4).

Lifestyle interventions remain the cornerstone of gestational diabetes mellitus (GDM) management. Upon diagnosis, pregnant women with GDM are advised to engage in at least 150 minutes of moderate-intensity physical activity each week. Additionally, they are encouraged to follow a diet that provides adequate macronutrients and micronutrients to support fetal growth, minimize postprandial glucose fluctuations, and promote appropriate gestational weight gain throughout pregnancy (12). Numerous randomized controlled trials (RCTs) and meta-analyses have demonstrated that exercise can delay the progression of glucose intolerance and improve maternal outcomes in women with GDM, leading to its recommendation for all women with GDM without contraindications to physical activity (13, 14). Despite robust evidence supporting the maternal benefits of prenatal exercise, there is limited research on its effects on neonatal outcomes. A recently published meta-analysis reported that exercise significantly reduces the rates of cesarean delivery, macrosomia, premature rupture of membranes, and neonatal hypoglycemia among women with GDM, but showed no impact on preterm birth rates (15). However, other adverse neonatal outcomes, such as intrauterine growth restriction and birth trauma, were not evaluated in this analysis. It is important to acknowledge that current literature often lacks detailed information regarding key exercise parameters, including the specific type of exercise, its duration, intensity, and the trimester(s) during which the interventions were implemented. This limits our ability to fully understand the nuanced relationship between exercise and pregnancy outcomes. The question of whether prenatal exercise truly benefits neonatal outcomes in women with GDM, and the extent to which these risks can be mitigated, remains insufficiently explored. Furthermore, many studies do not provide statistically normalized results adjusted for potential confounders such as maternal age, pre-pregnancy body weight, or weight gain during pregnancy, which could influence the observed outcomes. The potential development of adverse events such as gestational hypertension related to workload, type of exercise or other demographics of the volunteers is also often not reported. Moreover, evidence suggests that awareness of the potential benefits of exercise for fetal health is a key enabler of pregnant women’s participation in prenatal physical activity (16). In light of these considerations, we conducted a systematic review and meta-analysis to explore whether prenatal exercise can reduce the risk of additional adverse neonatal outcomes, such as preterm birth, intrauterine growth restriction, and birth trauma, specifically in women with a confirmed diagnosis of GDM.

## Method

### Data sources and search strategies

This meta-analysis was conducted following the Preferred Reporting Items for Systematic Reviews and Meta-Analyses (PRISMA) guidelines (1). The study was prospectively registered in the international database of systematic reviews (PROSPERO; registration number: CRD42023485375).

To identify relevant studies, we systematically searched English-language databases, including PubMed, EMBASE, the Cochrane Library, Web of Science, and Scopus, from their inception to September 9, 2023. Our search utilized a combination of terms targeting participants and interventions, such as: *gestational diabetes mellitus OR GDM OR gestational diabetes OR pregnancy-induced diabetes* AND *sports OR exercise OR activit* OR physical activit* OR exercise training*. Full details of the search strategy are provided in online **Supplementary Table S1**.

Additionally, we searched ClinicalTrials.gov, though no relevant studies were identified through this source. To ensure comprehensive coverage, we also manually reviewed the reference lists of included articles and incorporated any appropriate studies identified during this process.

### Study selection

Study selection Studies were deemed eligible for inclusion if they met the following criteria: participants were definitively diagnosed with GDM; a complete exercise intervention program was implemented, specifying exercise duration, frequency, and intensity; The criteria used to categorize exercise intensity (mild, moderate, high) were based on the guidelines provided by the American College of Sports Medicine (ACSM) and were defined as follows: Mild intensity was defined as 30-39% HRR or VO2R, Moderate intensity as 40-59% HRR or VO2R, and High intensity as 60-89% HRR or VO2R. When studies did not directly report HRR or VO2R, we used METs (Metabolic Equivalents) as an alternative, with Mild intensity corresponding to 1.5-3 METs, Moderate intensity to 3-6 METs, and High intensity to >6 METs. the control group did not undergo any exercise intervention; and at least one outcome of interest was reported, such as cesarean delivery, preterm birth, or macrosomia. Studies with combined interventions (diet + exercise) were excluded because we aimed to isolate the specific effects of exercise on GDM outcomes and minimize confounding factors. The potential interaction between diet and exercise could obscure the true impact of exercise alone. Studies were excluded if they involved additional interventions beyond physical exercise, lacked original data (e.g., reviews, editorials, or comments), or were published in languages other than English. Study selection was conducted in two phases: an initial screening of titles and abstracts, followed by a full-text review of potentially eligible articles. Two independent reviewers (Hangyu Cui and Hua Li) assessed the eligibility of studies, with any discrepancies resolved by consultation with a third investigator (Mingzi Li).

### Risk of bias assessment

The risk of bias for intervention studies was assessed using the Cochrane Risk of Bias version 2 (RoB2) tool, following the guidelines outlined in the Cochrane Handbook. This tool evaluates five domains: bias arising from the randomization process, bias due to deviations from intended interventions, bias due to missing outcome data, bias in the measurement of outcomes, and bias in the selection of the reported results (2). For observational cohort studies, the Newcastle-Ottawa Scale (NOS) was employed to assess methodological quality.(3) The NOS evaluates eight categories related to study quality, with each study receiving a final score out of a maximum of nine points.Two authors (Hangyu Cui and Hua Li) independently conducted the evaluations, with any discrepancies resolved through discussion with a third investigator (Mingzi Li).

### Data extraction

Two authors (Hangyu Cui and Hua Li) independently extracted data from eligible studies using pretested, Excel-based data extraction sheets. The extracted information included the first author, publication year, study design, country, sample size, participants’ demographic characteristics, study setting, details of the intervention (type, frequency, intensity, and duration), and outcome variables of interest.

The adverse neonatal outcomes assessed in this study included cesarean delivery, macrosomia, preterm birth, neonatal complications, fetal growth restriction, birth trauma, low birth weight, neonatal hypoglycemia, premature rupture of membranes, large-for-gestational-age infants, gestational age at delivery, birth asphyxia, stillbirth, fetal distress, and congenital malformations.

### Data synthesis and analysis

Data analyses were performed using Stata Statistical Software version 18.0 (StataCorp, TX, USA) and R statistical software version 4.2.2. A p-value of <0.05 was considered statistically significant. Due to variations in study designs and the limited number of studies, a random-effects model was employed to calculate the estimated effect sizes and their respective 95% confidence intervals. Event counts and overall participant numbers were initially extracted, and odds ratios (ORs) were used for dichotomous variables. Heterogeneity across studies was assessed using the I² statistic, with thresholds of 0–25%, 25–50%, and >50% indicating low, moderate, and high heterogeneity, respectively. Sensitivity analyses were conducted to test the robustness of the results by sequentially removing individual studies. Publication bias was evaluated using funnel plots (for datasets with ≥10 studies) and the results of Egger’s and Begg’s tests.Given the broad publication time span and varying study designs, subgroup analyses were conducted to explore subgroup differences and potential sources of heterogeneity. Studies were stratified by study design (RCTs or observational studies), economic level of the study location (developed or developing countries), publication year (before or after 2018), type of exercise program (single-component or multi-component), and exercise mode (aerobic, resistance, or mixed exercise). The p-value for differences between subgroups was calculated, with a threshold of <0.1 considered statistically significant.

## Results

### Search results and quality assessment

**Figure 1:**
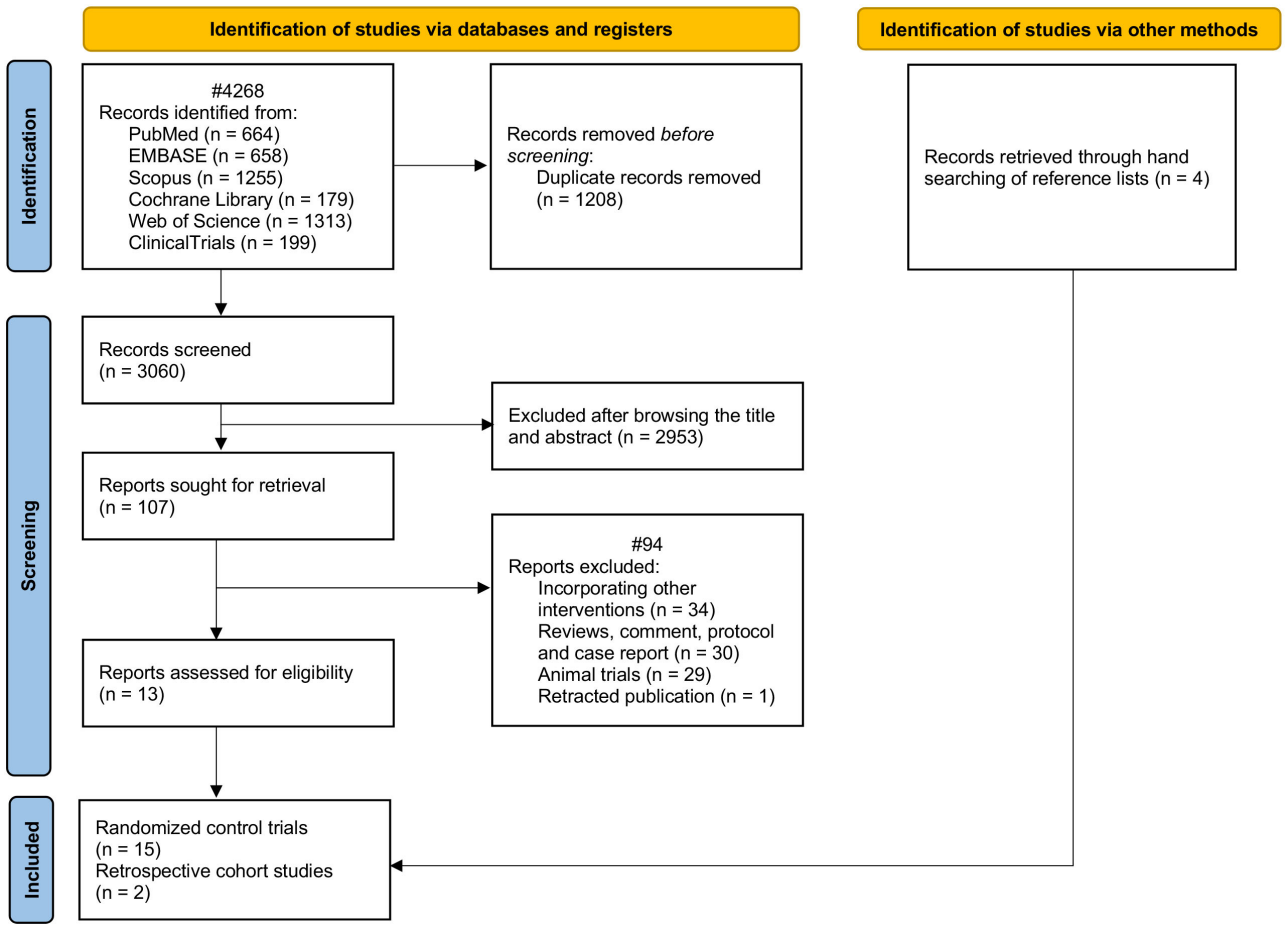
Flow chart of the study.

A total of 4,268 studies were identified through the database search. After removing duplicate records, 3,060 unique publications remained for the initial screening. Based on a review of titles and abstracts, 2,953 studies were excluded. Subsequently, a full-text review of the remaining articles led to a final inclusion of 17 studies (13 identified through database searches and 4 through manual searching). These included 15 randomized controlled trials (RCTs) (4–18) and 2 retrospective cohort studies (**Figure 1**) (19, 20). According to the RoB2 assessment, for studies analyzed using the intention-to-treat approach, 1 study (25%) demonstrated a low risk of bias, 2 studies (50%) had some concerns, and 1 study (25%) indicated a high risk of bias (online **Supplementary Figure S1**). When using the per-protocol analysis, 1 study (9.1%) showed a low risk of bias, 9 studies (81.8%) demonstrated some concerns, and 1 study (9.1%) indicated a high risk of bias. The two observational studies were of high quality, receiving scores of 8 and 9 points, respectively, on the Newcastle-Ottawa Scale (online **Supplementary Table S2**).

Characteristics of enrolled studies are presented in **Table 1**. 2200 and 77910 women with GDM were consisted in the 15 RCTs and 2 cohort studies, respectively. Exercise types comprised aerobic exercise, resistance exercise and composited exercise program. Duration of exercise ranged from 15 to more than 60 minutes with a frequency fluctuating between 2 and 7 times per week. The adverse pregnancy outcomes which were assessed in at least 2 publications so as to be pooled contained premature birth, macrosomia, cesarean section, fetal growth restriction, birth trauma, neonatal hypoglycemia, low birth weight, premature rupture of membranes, large for gestational age, birth asphyxia, stillbirth, fetal distress and congenital malformation.

**Table 1:** Characteristics of included studies.

Included literature	Country	Study design	Sample size		Intervention/Exposure information				Adverse neonatal outcomes^b^
			Experimental group/ Exposure group	Control group	Movement content	Frequency	Intensity^a^	Time (min)	
Eman (2019) (23)	Egypt	RCT	30	30	Circuit resistance training	3-4 times per week	Moderate	60	1
Ruben (2013) (25)	Spain	RCT	210	218	Resistance and aerobic exercises	3 times per week	Moderate	50-55	1
Jin (2022) (28)	China	RCT	65	66	Original gymnastics	10 times per week	Moderate	15	1, 2, 3, 6, 8, 13
Iva (2018) (29)	Croatia	RCT	18	20	Aerobic exercise resistance exercises, pelvic floor and stretching exercises	2 times per week	Moderate	30	1, 6
Vijay (2023) (30)	India	RCT	112	112	Walking, stationary biking, pelvic tilt, side plank, squats, breathing exercise and yoga	NA	NA	NA	1, 2, 3, 8, 12
Wu (2022) (32)	China	RCT	68	70	Walking, climbing stairs, jogging, cycling, pregnant yoga, pregnant gymnastics, swimming, lifting dumbbells	5 times per week	Moderate	30	1, 3, 6, 8
Xie (2021) (33)	China	RCT	43	46	Extension exercise	3 times per week	Moderate	50-60	1, 2, 3, 6, 8, 10
Zhao (2022) (34)	China	RCT	43	46	Resistance exercise of upper and lower limb muscles	3 times per week	Moderate	50-60	1, 2, 3, 6, 8, 10, 12
Abirami (2015) (20)	India	RCT	104	108	Yoga	Daily	NA	30-40	2, 3, 4, 5, 6, 10, 11
Raul (2007) (21)	America	RCT	39	57	Walking on a treadmill or by riding a semirecumbent cycle ergometer	6 days per week	Moderate	NA	1, 3, 7
Marcelo (2010) (27)	Brazil	RCT	32	32	Resistance training	3 nonconsecutive days a week	Moderate	NA	1, 2
Simona (2014) (26)	Italy	RCT	101	99	Brisk walking	Daily	Moderate	20	1, 2, 5, 9
Melissa (1997) (22)	America	RCT	15	14	Cycle ergometer, walking and cycling	3-4 times weekly	Moderate	30	1, 3
Balaji (2017) (24)	India	RCT	75	76	Yoga and pranayama	Daily	NA	60	2, 3, 4, 7, 11, 13
Carrie (2015) (31)	America	RCT	124	127	Dancing, walking, and yard work	Most days of the week	Moderate	at least 30	1, 2, 9
Carol (2008) (35)	America	Cohort	29480	45680	Aerobic exercise	3 times per week or more	Moderate	at least 30	1, 9
Wang (2015) (36)	China	Cohort	2061	689	NA	Daily	From mild to high	at least 60	1, 2, 3

a Mild: no work or sitting while working, walking less than 60 minutes a day; moderate: activities that require moderate physical effort and make a pregnant woman breathe a little harder than normal (such as cooking, sweeping the floor, washing clothes, average daily commute longer than 60 minutes or walking more than 60 minutes a day, carrying light loads, or bicycling at a regular pace); high: activities that require considerable physical effort and make a pregnant woman breathe much harder than normal (such as heavy lifting, aerobics, fast bicycling, dancing or swimming).

b 1. cesarean section; 2. premature birth; 3. macrosomia; 4. intra uterine growth restriction; 5. birth trauma; 6. neonatal hypoglycemia; 7. low birth weight infants; 8. premature rupture of membranes; 9. large for gestational age; 10. birth asphyxia; 11. stillbirth; 12. fetal distress; 13. congenital malformation.

### Association between exercise and adverse neonatal outcomes

**Figure 2:**
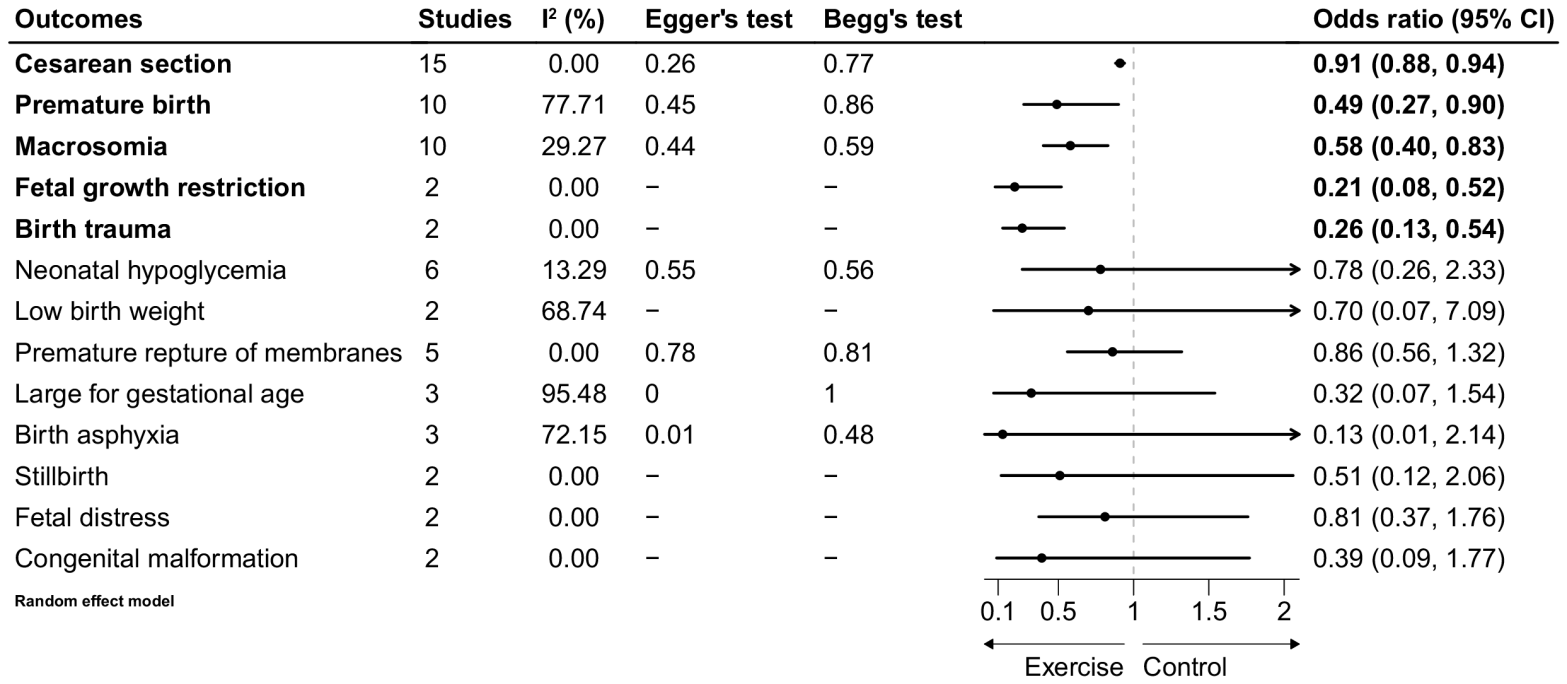
Forest plot of the association between exercise and selected adverse pregnancy outcomes.

The pooled results showed that exercise during pregnancy of women with GDM significantly decreased the incident rate of cesarean section (OR = 0.91, 95% CI: 0.88, 0.94), premature birth (OR = 0.49, 95% CI: 0.27, 0.90), macrosomia (OR = 0.58, 95% CI: 0.40, 0.83), fetal growth restriction (OR = 0.21, 95% CI: 0.08, 0.52), birth trauma (OR = 0.26, 95% CI: 0.13, 0.54). Heterogeneity of cesarean section (I^2^ = 0.00%), fetal growth restriction (I^2^ = 0.00%) and birth trauma (I^2^ = 0.00%) was all very low. Heterogeneity of macrosomia (I^2^ = 29.27%) was moderate, while that of premature birth (I^2^ = 77.71%) was substantial (**Figure 2**, **Supplementary Figures S2**-**S6**).

No significant association was observed between exercise during pregnancy and several neonatal outcomes in women with GDM. These outcomes included neonatal hypoglycemia (OR = 0.78, 95% CI: 0.26–2.33, P = 0.66), low birth weight (OR = 0.70, 95% CI: 0.07–7.09, P = 0.76), premature rupture of membranes (OR = 0.86, 95% CI: 0.27–2.74, P = 0.78), large-for-gestational-age infants (OR = 0.32, 95% CI: 0.07–1.54, P = 0.15), birth asphyxia (OR = 0.13, 95% CI: 0.01–2.14, P = 0.15), stillbirth (OR = 0.51, 95% CI: 0.12–2.06, P = 0.34), fetal distress (OR = 0.81, 95% CI: 0.37–1.76, P = 0.59), and congenital malformation (OR = 0.39, 95% CI: 0.09–1.77, P = 0.22) (**Figure 2**, **Supplementary Figures S7**-**S14**). Heterogeneity was low for congenital malformation, fetal distress, stillbirth, premature rupture of membranes, and neonatal hypoglycemia. In contrast, heterogeneity was high for birth asphyxia, large-for-gestational-age infants, and low birth weight (**Figure 2**).

### Sensitivity analysis and publication bias

The sensitivity analysis, performed by sequentially removing each study, confirmed the robustness of the pooled estimates for preterm birth, cesarean delivery, and macrosomia (online **Supplementary Figure S15**). However, the robustness of other outcomes was not assessed due to the limited number of studies or the lack of statistically significant associations. No evidence of publication bias was detected based on the visualization of funnel plots or the results of Egger’s and Begg’s tests (**Figure 2**, **Supplementary Figure S16**).

### Subgroup analysis

**Figure 3:**
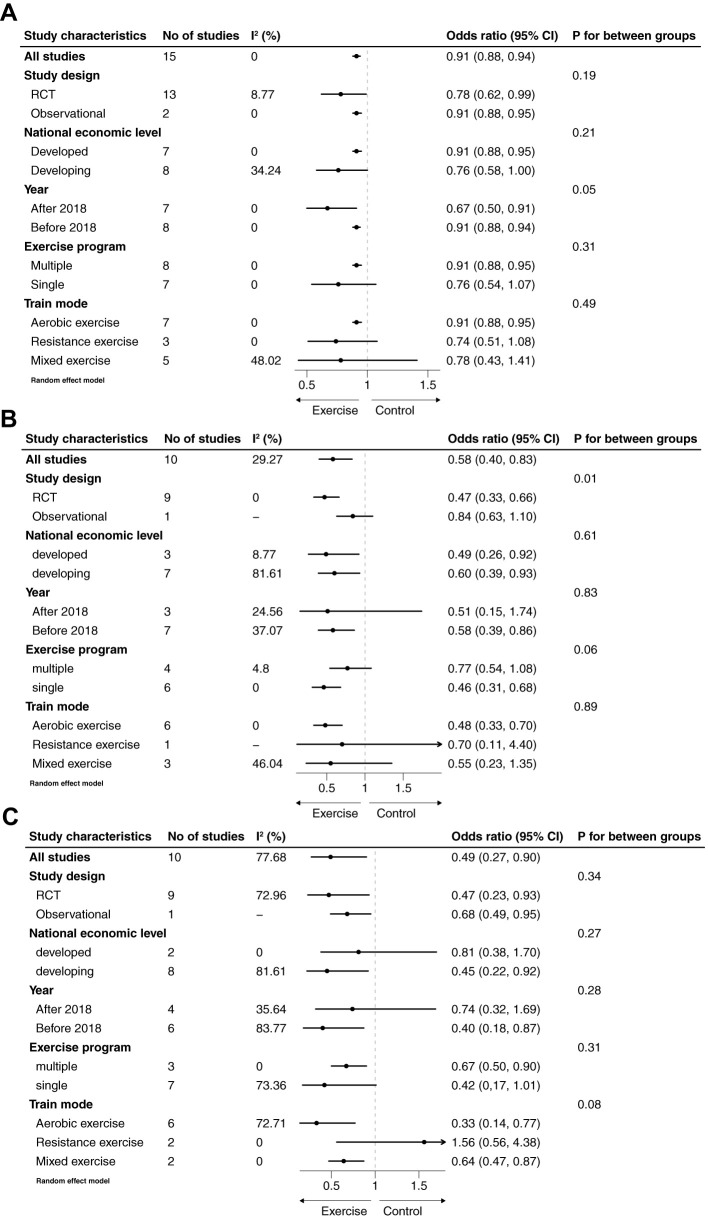
Subgroup analyses of association between exercise and cesarean section **(A)**, premature birth **(B)** and macrosomia **(C)**.

To examine whether the association between exercise and selected adverse pregnancy outcomes was influenced by subgroup differences, subgroup analyses were conducted, with a p-value of <0.1 considered indicative of potential interactions. Given the growing popularity of exercise interventions and improved exercise compliance in recent years, subgroup analyses were stratified by publication year. The results revealed a significant difference in the association between exercise and cesarean delivery based on the year of publication (P = 0.05). Studies published after 2018 demonstrated a more pronounced effect of exercise interventions on reducing cesarean delivery rates compared to those published earlier (**Figure 3A**). Additionally, there was a consistent trend indicating that single-component exercise programs were more effective in reducing the risk of cesarean delivery, preterm birth, and macrosomia compared to multi-component programs that combined different types of exercise. However, it is important to note that this conclusion requires caution, as no studies directly compared single-component and multi-component interventions within the same trial. This observed trend is based on indirect comparisons across different studies. Similarly, randomized controlled trials (RCTs) showed better results than observational studies, although statistical significance was observed only for macrosomia. Furthermore, subgroup analyses suggested that aerobic exercise specifically conferred benefits in reducing adverse neonatal outcomes (**Figure 3**).

## Discussion

This systematic review and meta-analysis included data from 17 studies, comprising 15 randomized controlled trials (RCTs) and 2 retrospective cohort studies. The findings indicate that exercise during pregnancy significantly reduces the risks of adverse pregnancy outcomes, including cesarean delivery, preterm birth, macrosomia, fetal growth restriction, and birth trauma. Notably, the analysis underscores the critical role of regular, sustained, and moderate-intensity exercise in mitigating these risks. Furthermore, single-component exercise programs demonstrated greater effectiveness in improving adverse neonatal outcomes compared to multi-component programs, emphasizing the value of targeted exercise interventions during pregnancy.

Premature birth and macrosomia are common neonatal complications among women with GDM, often accompanied by an increased incidence of cesarean delivery (21, 22). While it is widely acknowledged that exercise can delay the progression of glucose intolerance by enhancing insulin secretion and improving maternal outcomes for women with GDM, few studies have specifically investigated the association between exercise and adverse neonatal outcomes (23–25). Previous meta-analyses focusing on pregnant women, in general, reported that exercise reduced the risk of macrosomia, with risk reductions ranging from 4% to 61%. However, specific data concerning the impact of exercise on macrosomia in women with GDM remained unavailable (26, 27). A meta-analysis published in 2022, which included 9 studies, found that exercise reduced the incidence of macrosomia (RR = 0.57, P = 0.03) and cesarean delivery (RR = 0.83, P = 0.02), while outcomes such as preterm birth, premature rupture of membranes, and neonatal hypoglycemia were not significantly affected (28). Despite these findings, the relationship between exercise and certain adverse neonatal outcomes in women with GDM remains inconclusive, leaving significant gaps in understanding and a need for further research on this topic.

In our systematic review and meta-analysis, we comprehensively evaluated adverse neonatal outcomes associated with GDM. The pooled results demonstrated that exercise during pregnancy significantly reduced the incidence of macrosomia, cesarean delivery, preterm birth, fetal growth restriction, and birth trauma. Additionally, we explored the association of exercise with other adverse neonatal outcomes, including neonatal hypoglycemia, low birth weight, premature rupture of membranes, large-for-gestational-age infants, stillbirth, birth asphyxia, fetal distress, and congenital malformations. However, none of these associations were found to be statistically significant. This study offers several advantages over previous meta-analyses. First, it included a larger number of studies, enabling a comprehensive and systematic assessment of the effects of exercise on adverse neonatal outcomes in women with GDM, thereby providing a more holistic understanding of the association. Second, the results are robust and reliable, supported by the absence of publication bias and minimal heterogeneity across the included studies. Third, the study’s strengths include its large sample size and representation across diverse countries, enhancing the generalizability of the findings to broader populations.

In the subgroup analyses, the associations between exercise and specific adverse neonatal outcomes varied based on study design, publication year, exercise program, and exercise mode. The association between exercise and outcomes such as cesarean delivery, preterm birth, and macrosomia was attenuated to varying degrees in multiple-component exercise programs compared to single-component programs, although statistical significance was observed only for macrosomia (P = 0.06). Among the included studies, 7 implemented multiple-component exercise interventions, while 9 employed single-component interventions. Additionally, the retrospective study by Wang et al. did not provide details about the specific types of exercise involved; however, the exercise program was categorized as multiple-component due to the systematic cluster sampling method used to recruit participants (36). None of the included studies directly compared the effects of single- versus multiple-component exercise programs. To address this gap, we analyzed the differences by dividing the studies into two subgroups. A plausible explanation for the observed variation is that single-component exercise programs may be easier for pregnant women with GDM to adhere to. This is particularly relevant as the gravid uterus can obstruct the aorta and inferior vena cava, reducing cardiac output and potentially discouraging physical activity as pregnancy progresses (42–44). However, it’s also important to consider that differences in program adherence or participant characteristics, such as baseline fitness levels, motivation, or access to resources, could also contribute to this finding. For example, participants in single-component programs might have had higher overall adherence rates or may have been more motivated due to the simpler nature of the intervention. Further investigation is needed to determine whether these factors influenced the observed trend.

Different exercise modes also exhibited varying effects, with aerobic exercise specifically reducing the incidence of adverse neonatal outcomes. This aligns with current recommendations from the American College of Obstetricians and Gynecologists (ACOG), which advocate for regular aerobic exercise during pregnancy (45). However, a study comparing the effects of resistance versus aerobic exercise on blood glucose control found that resistance exercise was more effective in reducing 2-hour postprandial blood glucose levels (14). This difference may be attributed to the distinct physiological mechanisms by which aerobic and resistance exercise impact glucose metabolism. Aerobic exercise primarily enhances insulin sensitivity and glucose uptake in skeletal muscles through increased blood flow and energy expenditure. Resistance exercise, on the other hand, promotes muscle hypertrophy and increases basal metabolic rate, leading to improved long-term glucose control and insulin sensitivity. Furthermore, resistance exercise may directly stimulate glucose disposal independent of insulin, offering a unique benefit for managing GDM. It is important to interpret these findings with caution, as there is limited evidence directly comparing the effects of these two exercise modes. Further research is needed to clarify their relative benefits for pregnant women with GDM.

There was a consistent trend indicating that exercise had a greater impact on reducing adverse neonatal outcomes in randomized controlled trials (RCTs) compared to observational studies. This is likely because, in interventional studies, exercise is implemented through structured programs with scheduled activities and timely guidance, ensuring better adherence and standardized practices (29). Additionally, the pooled effect of studies published before 2018 was weaker compared to those published in more recent years. This difference may reflect advancements in intervention strategies, with more rational and evidence-based approaches being adopted over time. Moreover, increased awareness and efforts to address perceived barriers to physical activity in pregnant women may have contributed to improved exercise compliance and outcomes in recent studies (30).

In summary, exercise during pregnancy serves as an effective intervention to reduce the incidence of various neonatal complications in women with GDM, without increasing the risk of adverse events (34, 48). The subgroup differences observed in this study suggest that simpler and more accessible forms of exercise yield greater benefits, highlighting the importance of tailoring exercise interventions to enhance adherence among pregnant women. Based on current guidelines and the findings of this review, we recommend that women with GDM engage in at least 150 minutes of moderate-intensity aerobic exercise per week, spread across at least three days. This could include activities such as brisk walking, swimming, or stationary cycling. Each session should ideally last for at least 30 minutes. Furthermore, the inclusion of resistance exercise, performed 2-3 times per week, can offer additional benefits for glucose control. However, it is crucial to emphasize the importance of professional supervision, especially for resistance exercises, to ensure proper form and technique, minimize the risk of injury, and optimize adherence. A qualified healthcare professional or certified exercise specialist can provide personalized guidance and monitor progress throughout the pregnancy. However, the potential risk of bias underscores the need for high-quality research to further clarify the association between exercise and neonatal outcomes. Future studies should focus on improving study design, ensuring broader geographical representation, and adequately controlling for confounding factors to address existing evidence gaps and provide more robust and generalizable conclusions.

This study has several limitations that should be acknowledged. First, as with all meta-analyses, the quality of the included studies inherently constrains the overall validity of the findings. While most of the RCTs included exhibited either some concerns or a high risk of bias, the low overall heterogeneity and absence of significant publication bias suggest that the results remain statistically reliable. Second, the two observational studies included in this analysis had relatively large sample sizes, which gave them greater weight in the pooled results and may have influenced the overall outcomes. However, through subgroup analyses and sensitivity testing—where individual studies were removed one by one—the pooled findings remained consistent and robust, supporting the reliability of the conclusions. Finally, the association between exercise and certain adverse neonatal outcomes was evaluated using only a limited number of studies. As a result, these findings should be interpreted with caution, and further research is required to validate these associations and strengthen the evidence base. Specifically, we acknowledge the limitations stemming from the lack of detailed information in the included studies regarding crucial aspects of the exercise interventions. We often lacked data on the specific type of exercise, duration, intensity, and the gestational trimester(s) of participation. This makes it difficult to determine the optimal exercise prescription for women with GDM. Furthermore, there was often a lack of information regarding statistical normalization for potential confounders like maternal age, pre-pregnancy BMI, or weight gain during pregnancy across the included studies. This absence of standardized reporting limits our ability to isolate the independent effect of exercise. Finally, we note that information regarding the development of adverse events, such as gestational hypertension related to exercise workload, type of exercise, or other demographics, was often missing. This prevents us from fully assessing the safety and tolerability of exercise interventions in this population. These data gaps highlight the need for future studies to incorporate more comprehensive and standardized reporting of exercise parameters, potential confounders, and adverse events.

## Conclusion

In conclusion, this systematic review and meta-analysis demonstrated that exercise, particularly single-component exercise programs, can significantly reduce the risk of adverse neonatal outcomes in women with GDM, including macrosomia, preterm birth, cesarean delivery, fetal growth restriction, and birth trauma. These findings emphasize the substantial benefits of antenatal exercise for fetal health, underscoring its importance as a cornerstone intervention for women diagnosed with GDM. To further enhance our understanding, long-term follow-up studies are warranted to evaluate the sustained effects of exercise interventions on the health and development of offspring. However, the conclusions should be interpreted with caution, considering the limitations regarding the quality of information, the normalization of results as well as a comprehensive detailing of the exercise programs.
