# Supplementary material for: The effect of exercise on the adverse neonatal outcomes related to women with gestational diabetes mellitus: a systematic review and meta-analysis
Source: Front Clin Diabetes Healthc. 2025 Apr 1;6:1566577. doi: 10.3389/fcdhc.2025.1566577 (PMC11997568; doi:10.3389/fcdhc.2025.1566577)
Supplement: Supplementary file 1 [file DataSheet1.pdf]

The Effect of Exercise on the Adverse Neonatal Outcomes Related to Women with Gestational Diabetes Mellitus: A Systematic Review and Meta-analysis

Supplementary Material

Content

Table S1. Details of the search strategy. 2

Table S2. Quality assessment of the observational studies with NOS scale. 5

Figure S1. Risk of Bias summary for included studies which applied intention-to-treat analysis (A). Risk of Bias summary for included studies which applied per-protocol analysis (B). 6

Figure S2. Forest plot of association between exercise and cesarean section in women with GDM. 7

Figure S3. Forest plot of association between exercise and premature birth in women with GDM. 8

Figure S4. Forest plot of association between exercise and macrosomia in women with GDM. . 9

Figure S5. Forest plot of association between exercise and fetal growth restriction in women with GDM. 10

Figure S6. Forest plot of association between exercise and birth trauma in women with GDM. 11

Figure S7. Forest plot of association between exercise and neonatal hypoglycemia in women with GDM. 12

Figure S8. Forest plot of association between exercise and low birth weight infants in women with GDM. 13

Figure S9. Forest plot of association between exercise and premature rupture of membranes in women with GDM. 14

Figure S10. Forest plot of association between exercise and large for gestational age in women with GDM. 15

Figure S11. Forest plot of association between exercise and birth asphyxia in women with GDM. 16

Figure S12. Forest plot of association between exercise and stillbirth in women with GDM. ... 17

Figure S13. Forest plot of association between exercise and fetal distress in women with GDM. 18

Figure S14. Forest plot of association between exercise and congenital malformation in women with GDM. 19

Figure S15. Sensitivity analysis by removing eligible studies one by one, (A) cesarean section, (B) premature birth and (C) macrosomia. 20

Figure S16. Funnel plot of (A) cesarean section, (B) premature birth and (C) macrosomia. 21

Table S1. Details of the search strategy.

PubMed (664 publications)
(((((((((diabetes,gestational[MeSH Terms]) OR ("diabetes,gestational"[Title/Abstract])) OR ("Diabetes, Pregnancy-Induced"[Title/Abstract])) OR ("Diabetes, Pregnancy Induced"[Title/Abstract])) OR ("Pregnancy-Induced Diabetes"[Title/Abstract])) OR ("Gestational Diabetes"[Title/Abstract])) OR ("Diabetes Mellitus, Gestational"[Title/Abstract])) OR ("Gestational Diabetes Mellitus"[Title/Abstract])) OR (GDM[Title/Abstract])) AND (Search: ((((((((((((((exercise[MeSH Terms]) OR (sports[MeSH Terms])) OR ("sedentary behavior"[MeSH Terms])) OR (Exercise*[Title/Abstract])) OR ("physical activit*"[Title/Abstract])) OR ("Activities, Physical"[Title/Abstract])) OR ("exercise,physical"[Title/Abstract])) OR ("exercises, physical"[Title/Abstract])) OR ("Physical exercise*"[Title/Abstract])) OR (Sport*[Title/Abstract])) OR (Athletic*[Title/Abstract])) OR ("Behavior, Sedentary"[Title/Abstract])) OR ("Sedentary Behavior*"[Title/Abstract])) OR ("Sedentary Lifestyle"[Title/Abstract])) OR ("Lifestyle, Sedentary"[Title/Abstract])) OR ("Physical Inactivity"[Title/Abstract]))) AND (Search: ((((((((((((randomized controlled trials as topic[MeSH Terms]) OR (Controlled Clinical Trial[MeSH Terms])) OR ("Controlled Clinical Trials as Topic"[MeSH Terms])) OR ("random allocation"[MeSH Terms])) OR ("Clinical Trials as Topic"[MeSH Terms])) OR ("randomized controlled trial*"[Title/Abstract])) OR ("Controlled Clinical Trial*"[Title/Abstract])) OR ("Clinical Trials, Randomized"[Title/Abstract]) OR ("Trials, Randomized Clinical"[Title/Abstract]) OR ("Controlled Clinical Trials, Randomized"[Title/Abstract]) OR ("Clinical Trials, Controlled"[Title/Abstract])) OR ("Clinical Trial"[Title/Abstract])) OR (Group[Title/Abstract])) OR (Randomized[Title/Abstract])) OR (Randomly[Title/Abstract])) OR (Trial[Title/Abstract]))
**Embase (658 publications)**
#1 gestational diabetes'/exp OR 'diabetes mellitus gravidarum':ab,ti OR 'diabetes mellitus of pregnancy':ab,ti OR 'diabetes of pregnancy':ab,ti OR 'diabetes, gestational':ab,ti OR 'diabetes, pregnancy':ab,ti OR 'gestational diabetes mellitus':ab,ti OR 'maternal gestational diabetes mellitus':ab,ti OR 'pregnancy diabetes':ab,ti OR 'pregnancy diabetes mellitus':ab,ti OR 'pregnancy-induced diabetes':ab,ti OR 'gestational diabetes':ab,ti<br>#2 'exercise'/exp OR 'sport'/exp OR 'sedentary lifestyle'/exp OR 'exercise training':ab,ti OR 'fitness training':ab,ti OR 'physical exercise':ab,ti OR exercise:ab,ti OR sport*:ab,ti OR 'sedentary behavior':ab,ti OR 'sedentary behaviour':ab,ti OR 'sedentary life style':ab,ti OR 'sedentary lifestyle':ab,ti<br>#3 'randomized controlled trial'/exp AND topic OR ('clinical trial'/exp AND topic) OR 'randomization'/exp OR ('controlled clinical trial'/exp AND topic) OR 'randomized controlled trial*':ab,ti OR 'clinical trial*':ab,ti OR group:ab,ti OR 'random allocation':ab,ti OR randomi*ation:ab,ti OR 'controlled clinical trial*'<br>#1AND#2AND#3

**Web of Science (1313 publications)**
#1 (((((((((((((((TS=("diabetes, gestational")) OR TI=("diabetes, gestational")) OR TS=("Diabetes, Pregnancy-Induced")) OR TI=("Diabetes, Pregnancy-Induced")) OR TI=("Diabetes, Pregnancy Induced")) OR TS=("Diabetes, Pregnancy Induced")) OR TS=("Pregnancy-Induced Diabetes")) OR TI=("Pregnancy-Induced Diabetes")) OR TS=("Gestational Diabetes")) OR TI=("Gestational Diabetes")) OR TS=("Diabetes Mellitus, Gestational")) OR TI=("Diabetes Mellitus, Gestational")) OR TS=("Gestational Diabetes Mellitus")) OR TI=("Gestational Diabetes Mellitus")) OR TS=(GDM)) OR TI=(GDM)<br>#2 (((((((((((((((((((((((((TS=(exercise*)) OR TI=(exercise*)) OR TI=(sport*)) OR TS=(sport*)) OR TS=("sedentary behavior*")) OR TI=("Sedentary behavior*")) OR TS=("Physical activit*")) OR TI=("Physical activit*")) OR TS=("Activities, Physical")) OR TI=("Activities, Physical")) OR TS=("Exercise, physical")) OR TI=("Exercise, physical")) OR TS=("exercises, physical")) OR TI=("exercises, physical")) OR TS=("Physical exercise*")) OR TI=("Physical exercise*")) OR TS=(Athletic*)) OR TI=(Athletic*)) OR TS=("Behavior, Sedentary")) OR TI=("Behavior, Sedentary")) OR TS=("Sedentary Lifestyle")) OR TI=("Sedentary Lifestyle")) OR TS=("Lifestyle, Sedentary")) OR TI=("Lifestyle, Sedentary")) OR TS=("Physical Inactivity")) OR TI=("Physical Inactivity")<br>#3 (((((((((((((((((((((((TS=("Random allocation")) OR TI=("Random allocation")) OR TS=("Randomized controlled trial*")) OR TI=("Randomized controlled trial*")) OR TS=("Controlled Clinical Trial*")) OR TI=("Controlled Clinical Trial*")) OR TS=("Clinical Trials, Randomized")) OR TI=("Clinical Trials, Randomized")) OR TS=("Trials, Randomized Clinical")) OR TI=("Trials, Randomized Clinical")) OR TS=("Controlled Clinical Trials, Randomized")) OR TI=("Controlled Clinical Trials, Randomized")) OR TS=("Clinical Trials, Controlled")) OR TI=("Clinical Trials, Controlled")) OR TS=("Clinical Trial")) OR TI=("Clinical Trial")) OR TS=(Group)) OR TI=(Group)) OR TS=(Randomized)) OR TI=(Randomized)) OR TS=(Randomly)) OR TI=(Randomly)) OR TS=(Trial)) OR TI=(Trial)<br>#1AND#2AND#3
**Scopus (1255 publications)**
#1 TITLE-ABS-KEY ("Gestational Diabetes Mellitus") OR TITLE-ABS-KEY ("Diabetes, Pregnancy-Induced") OR TITLE-ABS-KEY ("Diabetes, Pregnancy Induced") OR TITLE-ABS-KEY ("Diabetes, Pregnancy Induced") OR TITLE-ABS-KEY ("Gestational Diabetes") OR TITLE-ABS-KEY ("Diabetes Mellitus, Gestational") OR TITLE-ABS-KEY ("Gestational Diabetes Mellitus") OR TITLE-ABS-KEY ("GDM")<br>#2 TITLE-ABS-KEY ("Exercise*") OR TITLE-ABS-KEY ("physical activit*") OR TITLE-ABS-KEY ("Activities, Physical") OR TITLE-ABS-KEY ("Activity, Physical") OR TITLE-ABS-KEY ("exercise, physical") OR TITLE-ABS-KEY ("exercises, physical") OR TITLE-ABS-KEY ("Physical exercise*") OR TITLE-ABS-KEY ("Sport*") OR TITLE-ABS-KEY ("Athletic*") OR TITLE-ABS-KEY ("Behavior, Sedentary") OR TITLE-ABS-KEY ("Sedentary Behavior*") OR TITLE-ABS-KEY ("Sedentary Lifestyle") OR TITLE-ABS-KEY ("Lifestyle, Sedentary") OR TITLE-ABS-KEY ("Physical Inactivity")<br>#3 TITLE-ABS-KEY ("randomized controlled trial*") OR TITLE-ABS-KEY ("Controlled Clinical Trial**") OR TITLE-ABS-KEY ("Clinical Trials, Randomized") OR TITLE-ABS-KEY ("Trials, Randomized Clinical") OR TITLE-ABS-KEY ("Controlled Clinical Trials, Randomized") OR TITLE-ABS-KEY ("Clinical Trials, Controlled") OR TITLE-ABS-KEY

("Clinical Trial ") OR TITLE-ABS-KEY ("Group") OR TITLE-ABS-KEY ("Randomized") OR TITLE-ABS-KEY ("Randomly") OR TITLE-ABS-KEY ("Trial")<br>#1AND#2AND#3
**Cochrane library (179 publications)**
#1 MeSH descriptor: [Diabetes, Gestational] explode all trees<br>#2 (Diabetes, pregnancy-induced):ti,ab,kw OR (gestational diabetes):ti,ab,kw OR (pregnancy-induced diabetes):ti,ab,kw OR (gestational diabetes mellitus):ti,ab,kw OR (diabetes mellitus, gestational):ti,ab,kw<br>#3 MeSH descriptor: [Exercise] explode all trees<br>#4 MeSH descriptor: [Sports] explode all trees<br>#5 MeSH descriptor: [Sedentary Behavior] explode all trees<br>#6 (exercise*, physical):ti,ab,kw OR (physical activit*):ti,ab,kw OR (acute exercise*):ti,ab,kw OR (exercise training*):ti,ab,kw OR (sport):ti,ab,kw<br>#7 MeSH descriptor: [Randomized Controlled Trials as Topic] explode all trees<br>#8 MeSH descriptor: [Controlled Clinical Trials as Topic] explode all trees<br>#9 MeSH descriptor: [Random Allocation] explode all trees<br>#10 MeSH descriptor: [Clinical Trials as Topic] explode all trees<br>#11 (controlled clinical trials, randomized):ti,ab,kw OR (clinical trials, randomized):ti,ab,kw OR (trials, randomized clinical):ti,ab,kw OR (clinical trials, controlled as topic):ti,ab,kw OR (randomization):ti,ab,kw with Cochrane Library publication date to Sep 2023<br>#12 (#1 OR #2) AND (#3 OR #4 OR #5 OR #6) AND (#7 OR #8 OR #9 OR #10 OR #11)
**ClinicalTrials (199 trials)**
Condition or disease: Gestational Diabetes Mellitus<br>Other terms: exercise

Table S2. Quality assessment of the observational studies with NOS scale.

Study	Selection				Comparability		Outcome			Total score
	Representative-ness	Selection of non-exposed	Ascertainment of exposure	Outcome not present at start	Comparability on most important factors	Comparability on other risk factors	Assessment of outcome	Long enough follow-up	Adequacy of follow-up	
Carol, et al (2008)	1	1	1	1	1	1	1	1	1	9
Wang, et al (2015)	1	1	1	1	1	0	1	1	1	8

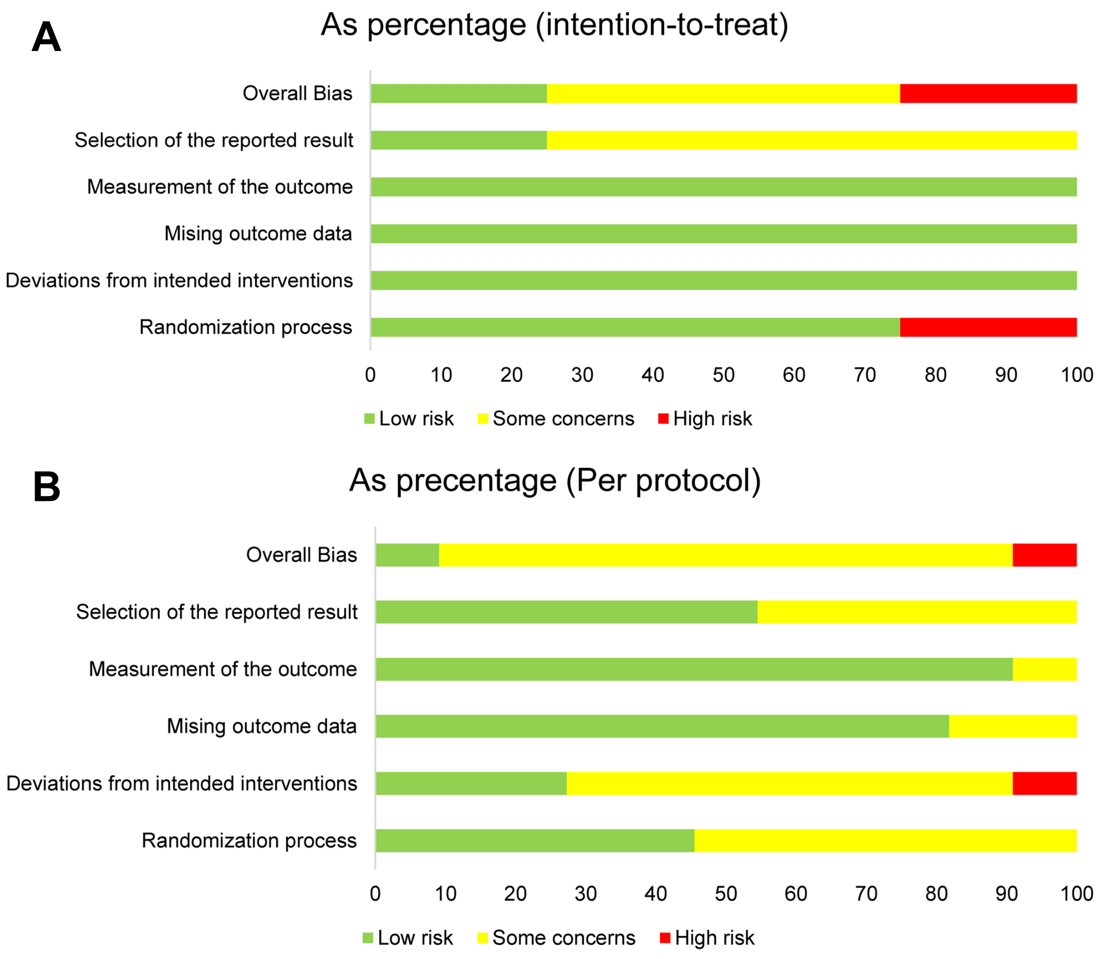

Figure S1. Risk of Bias summary for included studies which applied intention-to-treat analysis (A). Risk of Bias summary for included studies which applied per-protocol analysis (B).

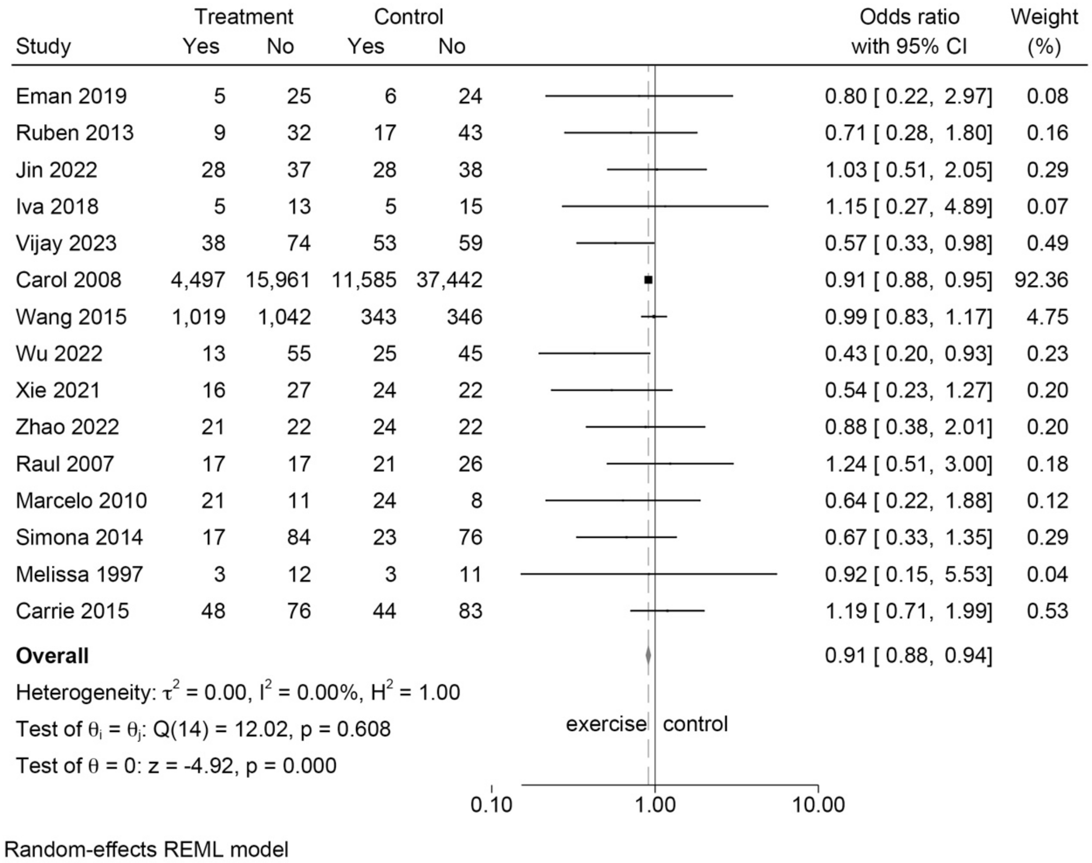

Figure S2. Forest plot of association between exercise and cesarean section in women with GDM.

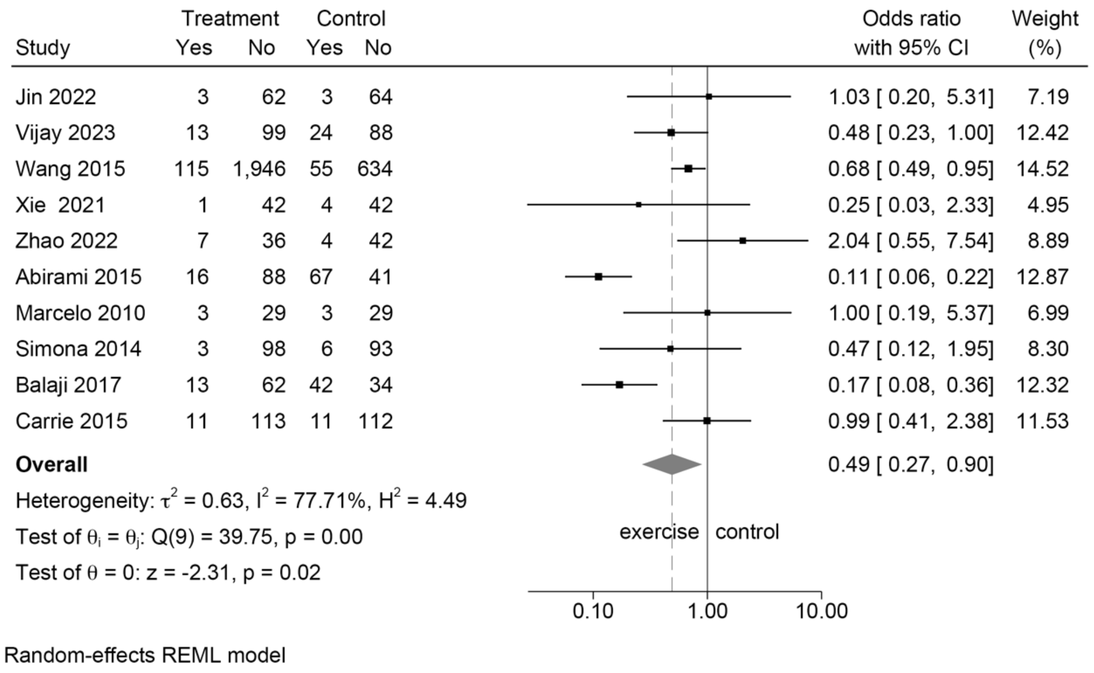

Figure S3. Forest plot of association between exercise and premature birth in women with GDM.

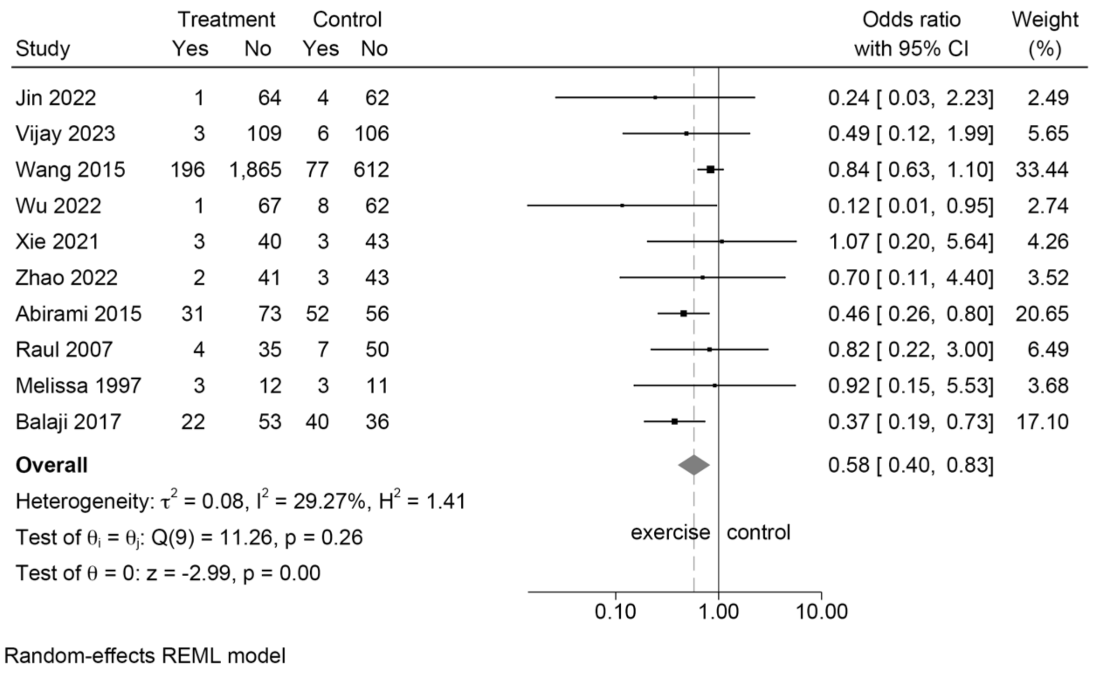

Figure S4. Forest plot of association between exercise and macrosomia in women with GDM.

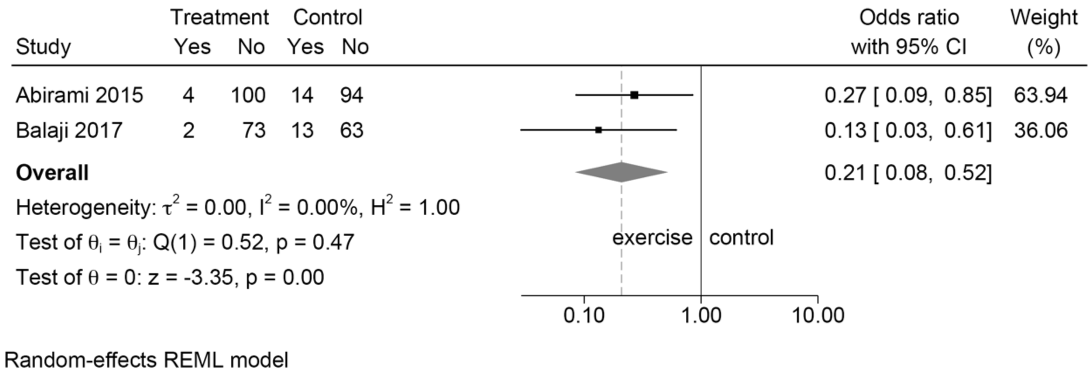

Figure S5. Forest plot of association between exercise and fetal growth restriction in women with GDM.

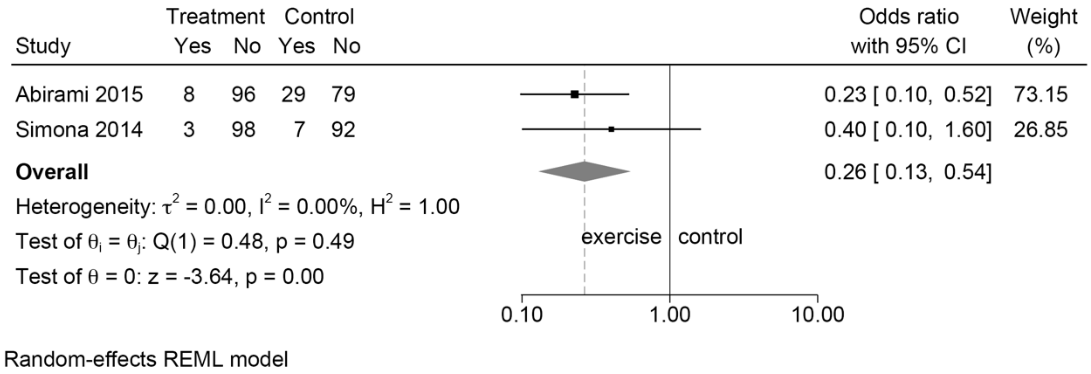

Figure S6. Forest plot of association between exercise and birth trauma in women with GDM.

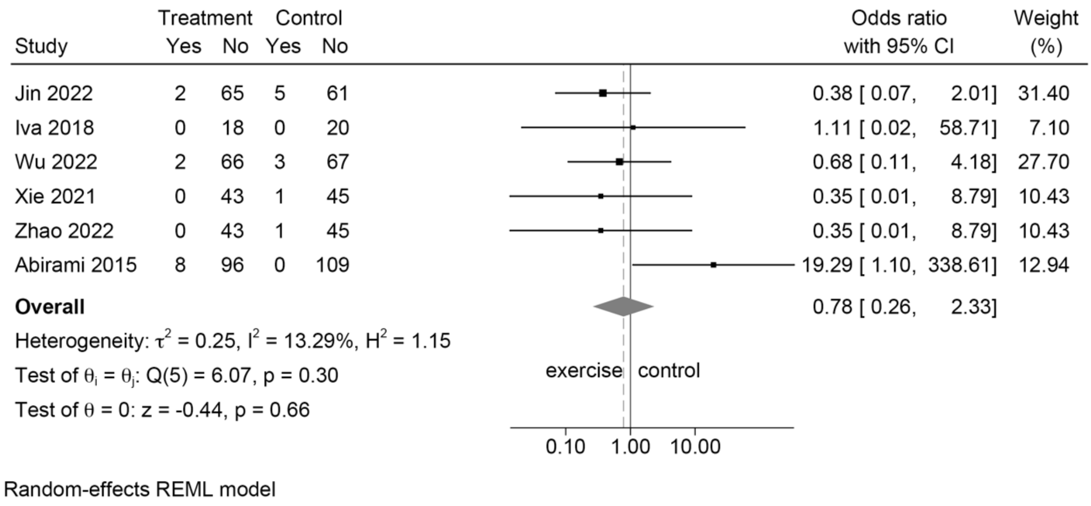

Figure S7. Forest plot of association between exercise and neonatal hypoglycemia in women with GDM.

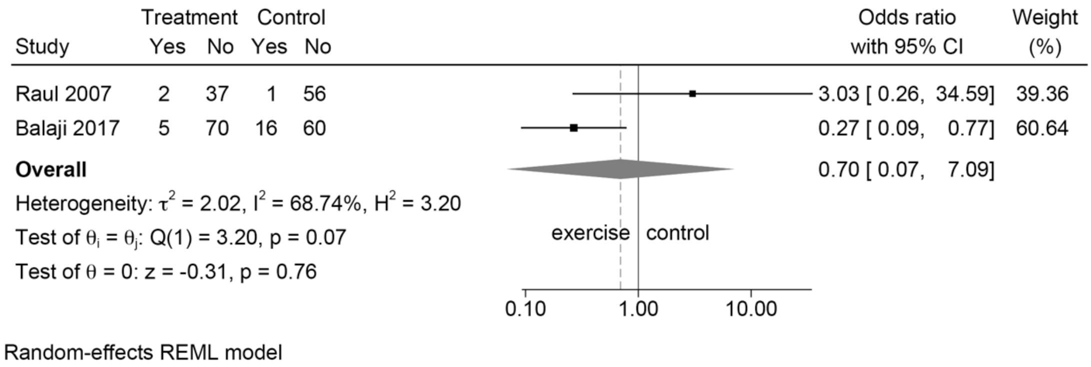

Figure S8. Forest plot of association between exercise and low birth weight infants in women with GDM.

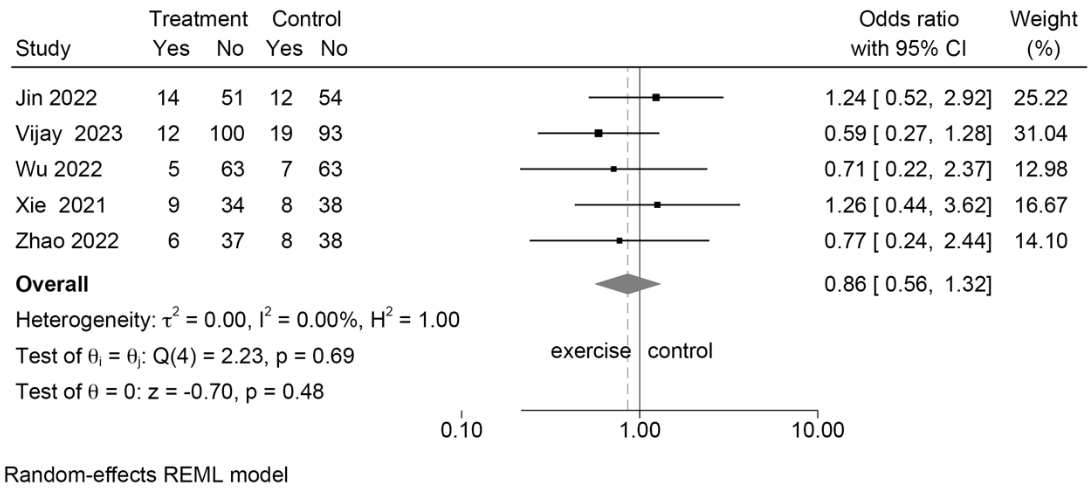

Figure S9. Forest plot of association between exercise and premature rupture of membranes in women with GDM.

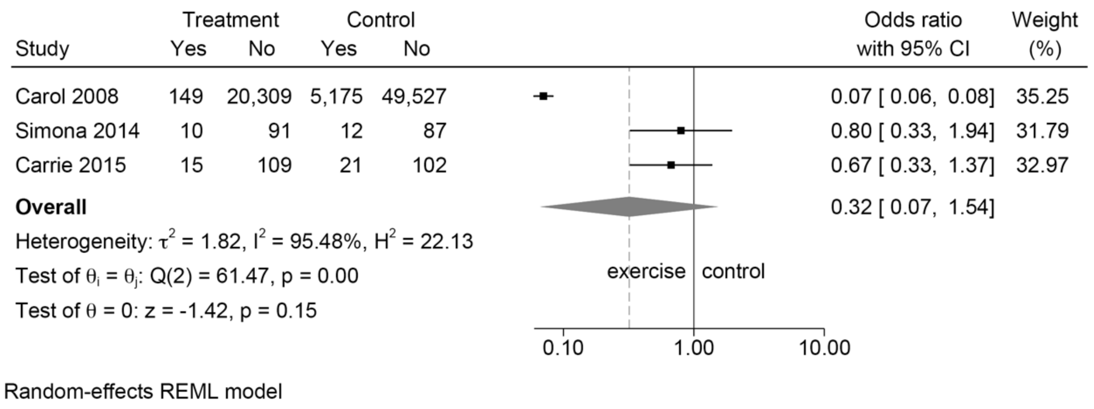

Figure S10. Forest plot of association between exercise and large for gestational age in women with GDM.

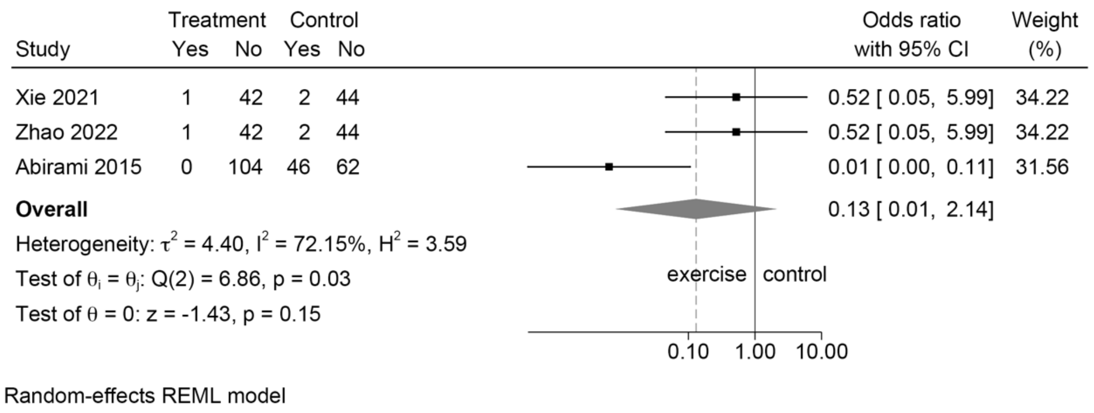

Figure S11. Forest plot of association between exercise and birth asphyxia in women with GDM.

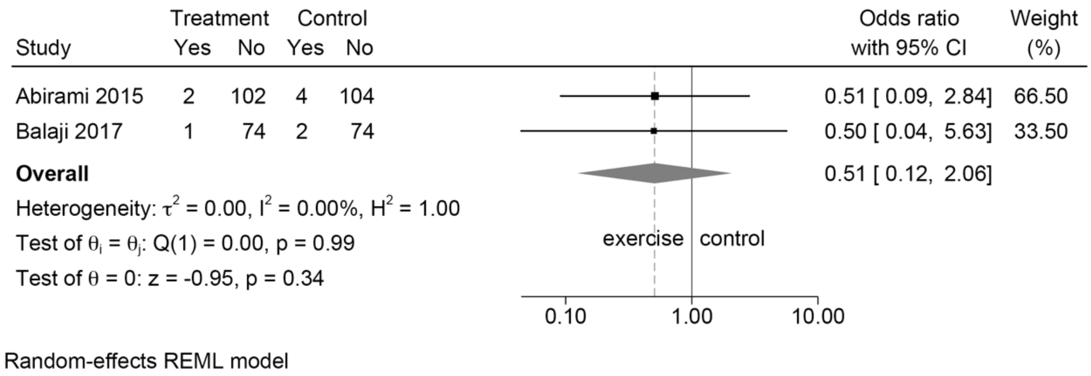

Figure S12. Forest plot of association between exercise and stillbirth in women with GDM.

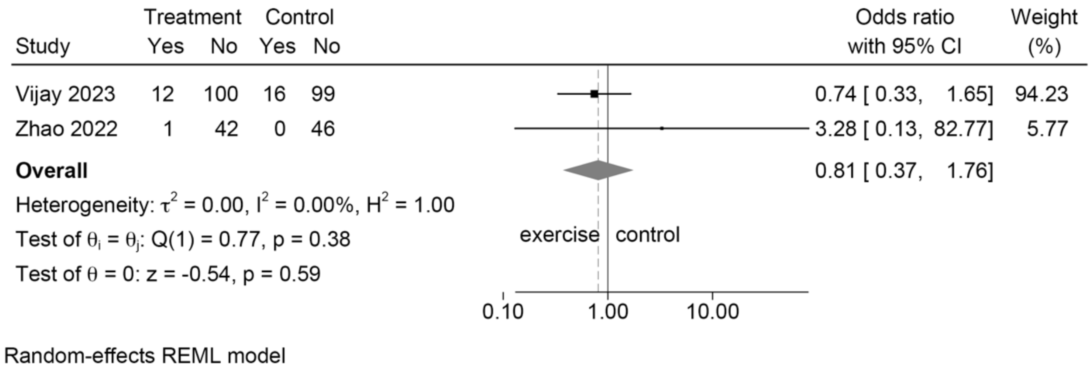

Figure S13. Forest plot of association between exercise and fetal distress in women with GDM.

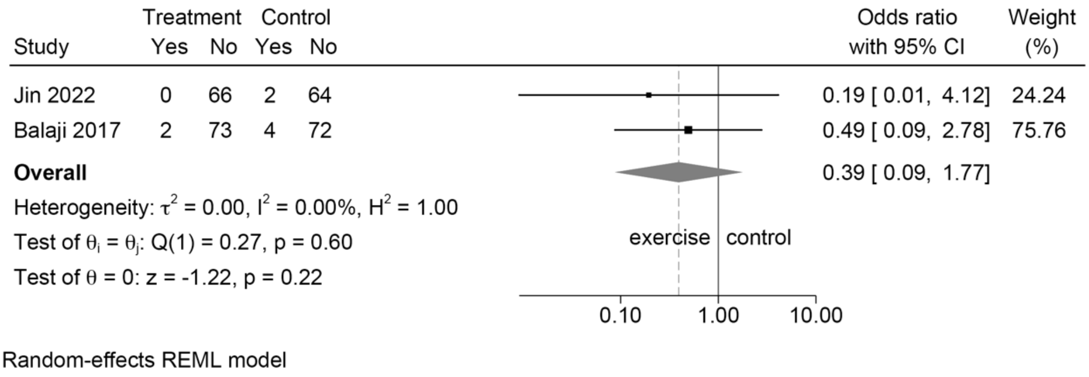

Figure S14. Forest plot of association between exercise and congenital malformation in women with GDM.

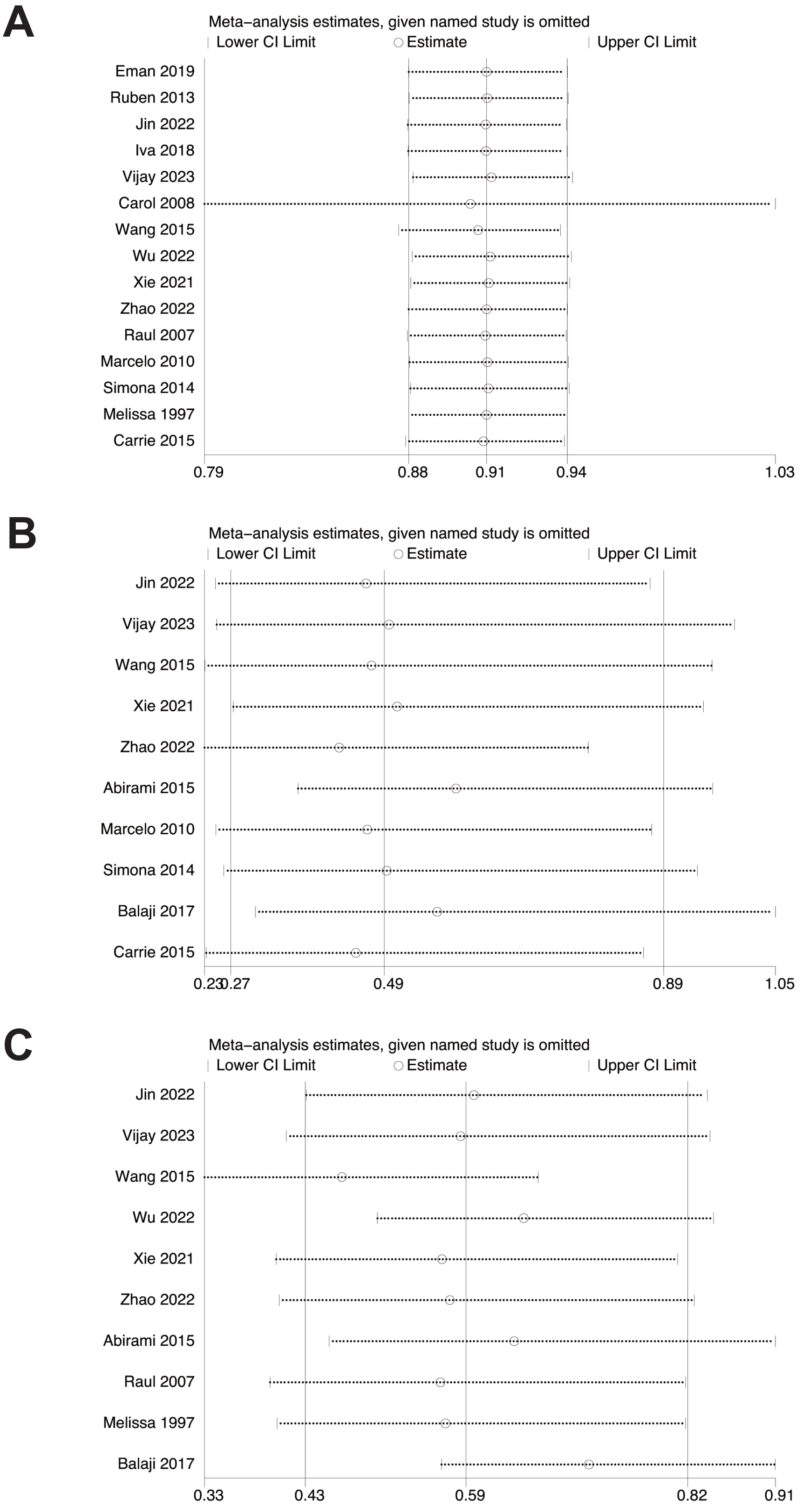

Figure S15. Sensitivity analysis by removing eligible studies one by one, (A) cesarean section, (B) premature birth and (C) macrosomia.

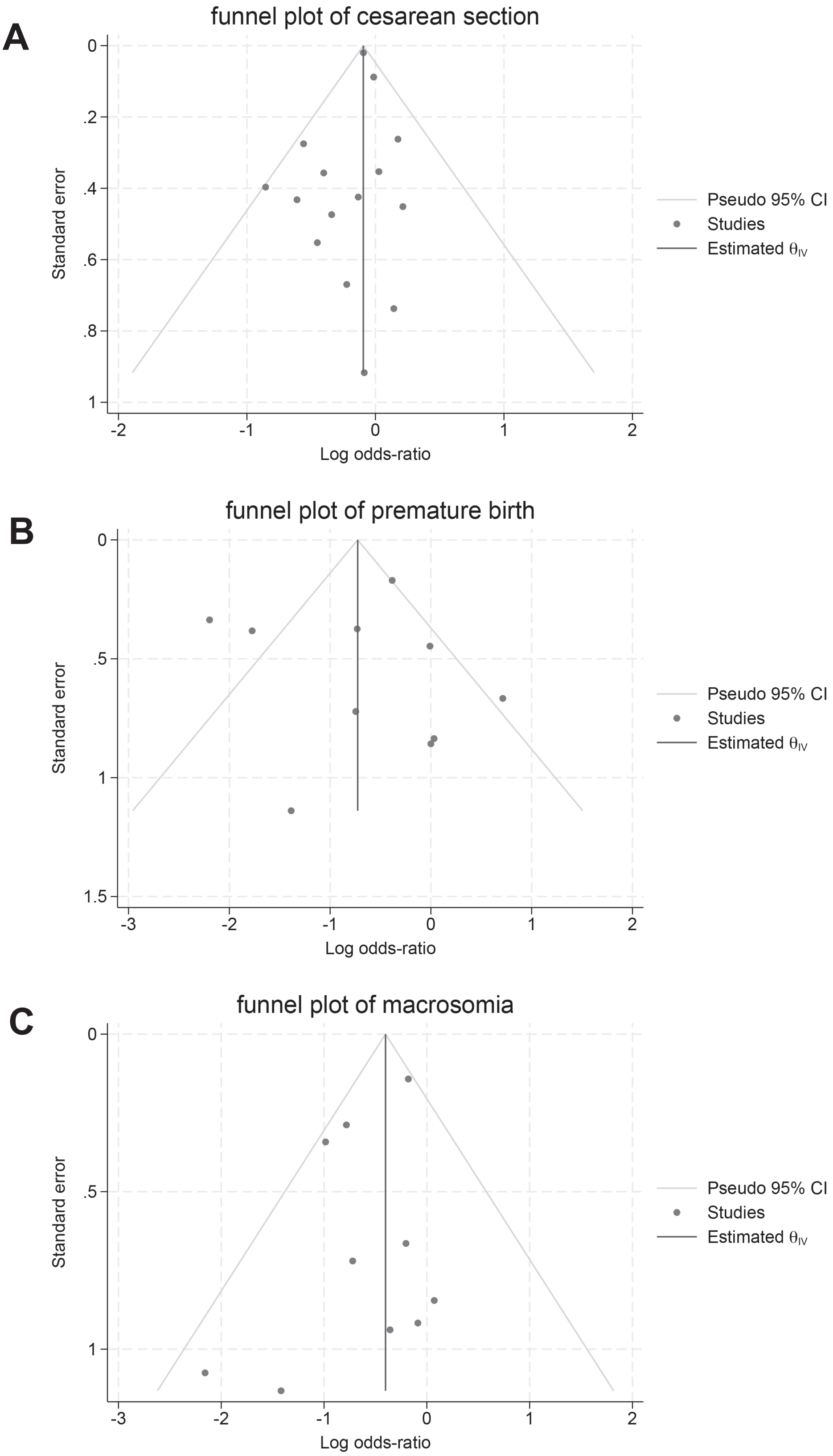

Figure S16. Funnel plot of (A) cesarean section, (B) premature birth and (C) macrosomia.
